# Licorice infusion: Chemical profile and effects on the activation and the cell cycle progression of human lymphocytes

**DOI:** 10.4103/0973-1296.59963

**Published:** 2010-02-13

**Authors:** José Cheel, Gabriela Onofre, Doris Vokurkova, Lenka Tůmová, Jarmila Neugebauerová

**Affiliations:** *Department of Pharmacognosy, Faculty of Pharmacy, Charles University, Heyrovského 1203, 500 05 Hradec Králové, Czech Republic*; 1 *Institute of Clinical Immunology and Allergology, Charles University, Faculty of Medicine and University Hospital, Sokolská 581, 500 05 Hradec Králové, Czech Republic*; 2 *Mendel University in Brno, Faculty of Horticulture, Valtická 337, 69144 Lednice, Czech Republic*

**Keywords:** CD69 expression, flavonoids, HPLC, licorice, lymphocytes, phenolics

## Abstract

A licorice infusion (LI) and its major constituents were investigated for their capacity to stimulate the activation and the cell cycle progression of human lymphocytes, measured by the CD69 expression and DNA content, respectively. The chemical profile of LI was determined by high-performance liquid chromatography-diode array detection (HPLC-DAD). Results: Two major components of LI were identified as liquiritin (1) and glycyrrhizin (2); total flavones and flavonols were shown as its minor constituents. The LI (100-800 μg/ml) stimulated the expression of CD69 on lymphocytes in a concentration-independent manner. Values of the activation index (AI) of total lymphocytes treated with LI (100-800 μg/ml) did not differ significantly among them (*P* < 0.05), but were 50% lower than the AI value exhibited by cells treated with phytohemagglutinin (PHA). The LI showed a similar effect on T cells, but on a lower scale. Compounds 1 and 2 (12-100 μg/ml) did not stimulate the CD69 expression on lymphocytes. The LI, 1 and 2 showed no meaningful effect on cell cycle progression of lymphocytes. The experimental data indicates that LI stimulates the activation of lymphocytes as a result of a proliferation-independent process. This finding suggests that LI could be considered as a potential specific immune stimulator.

## INTRODUCTION

Licorice, the dry roots of *Glycyrrhiza glabra* L. (Fabaceae), is considered one of the oldest and most widely used herbal drugs around the world, being present in most pharmacopoeias of Eastern and Western countries.[1] It has been traditionally used for respiratory, gastrointestinal, cardiovascular, genitourinary, eye, and skin disorders, and for its antiviral effects.[2] Glycyrrhizin and flavonoids such as liquiritin, isoliquiritin, and their aglycones have been reported as the major constituents of licorice and they are perceived as the active principles responsible for its pharmacological efficacy.[3]

The danger to global public health because of viral pandemic diseases such as those induced by influenza and HIV viruses requires the urgent evaluation of herbal drugs in widespread traditional use. Given that traditional sources mention licorice to treat symptoms attributable to viral infections, it is gaining attention as a potential immunomodulating agent.[4] The immunological action of herbs may involve the activation and induction of the cell cycle progression of immune cells, which play important roles in the generation of immune responses.[5] Licorice is consumed customarily in the form of teas and infusions,[6] but the immunomodulating properties of these aqueous preparations and the relation of such effect with its major constituents have been little explored.

The aim of the present study was to investigate the capacity of a licorice infusion (LI) and its major constituents to stimulate the activation and the cell cycle progression of human lymphocytes, using flow cytometry. The chemical profile of LI was determined by HPLC-DAD and spectrophotometric methods.

## MATERIALS AND METHODS

### Chemicals

Hide powder, standards (glycyrrhizin and quercetin), propidium iodide, ribonuclease A, phytohemagglutinin (PHA), and Tween-20 were obtained from Sigma Aldrich (Steinheim, Germany). Standard liquiritin was purchased from Wuhan Sunrise Technology Development Co., Ltd. (Hong Kong, China). Folin-Ciocalteu phenol reagent, aluminum chloride hexahydrate, gallic acid, and sodium carbonate were from Merck (Darmstadt, Germany). HPLC grade solvents were from Merck. Ultrapure water from the Milli-Q RG system (Millipore, Molsheim-France) was used. The monoclonal antibodies (phycoerythrin (PE), fluorescein isothiocyanate (FITC), and Allophycocyanin (APC) labeled) were obtained from Immunotech (France) and Dako (Denmark). X-Vivo medium was purchased from Bio-Wittaker (USA).

### Sample collection and infusion preparation

Roots of *G*. *glabra* were collected in February 2008 from the Botanical Garden of the Faculty of Horticulture, Mendel University in Brno, Czech Republic (situated 164 m above sea level). The genetic resource was identified with the code 0001. The plant material was dried at 40°C in an oven and was subsequently ground to fine powders (mesh size 20). The infusion was prepared by adding 150 ml of distilled water (95-100°C) to a precisely weighed amount (1.50 g) of licorice powder.[7] The infusion was brewed for 20 minutes and was then filtered over Whatman No. 1 paper. The resulting aqueous extract was lyophilized and the extraction yield was calculated based on the dry weight of the licorice. The licorice lyophilized infusion (LI) obtained was assessed for its biological activities and chemical profile.

### Determination of total content of phenolics

The total phenolic (TP) content was determined using the Folin-Ciocalteu procedure.[8] Briefly, the appropriate extract dilution was oxidized with the Folin-Ciocalteu reagent and the reaction was neutralized with sodium carbonate. The absorbance of the resulting blue color was measured at 760 nm after 30 minutes using a Shimadzu UV-1601 UV/ Vis spectrophotometer. Quantification was plotted on a standard curve of gallic acid. The results were expressed as mg gallic acid equivalents (GAE)/100 mg of LI. Data are reported as means ± standard deviation (SD) to an accuracy of three replicates.

### Determination of total content of tannins

After removal of tannins by adsorption on an insoluble matrix (hide powder), the total tannin (TT) content was determined by Folin-Ciocalteu procedure explained briefly in the previous paragraph. Calculated values were subtracted from the total phenolic contents and total tannin contents are expressed as mg gallic acid equivalents (GAE)/100 mg of LI. Data are reported as means ± standard deviation (SD) to an accuracy of three replicates.[9]

### Determination of total content of flavones and flavonols

The total flavones and flavonols (TF) content was determined according to the aluminum chloride method.[8] Quercetin was used as a reference for the calibration curve. The absorbance of the reaction mixture was measured at 415 nm. Results were expressed as mg quercetin equivalents (QE)/100 mg of LI. Data are reported as means ± SD to an accuracy of three replicates.

### HPLC- diode array detector analysis

The HPLC analysis was performed with a Jasco PU-2089 pump equipped with a Jasco MD-2015 diode array detector (DAD), and chromatographic separations were performed on a LiChrospher RP-18 column (4.0 × 250 mm i.d., 5 μm). Isocratic elution was used with a mobile phase containing acetonitrile: Methanol: Water: Glacial acetic acid (35:35:29:1, by volume) at a flow rate of 1 ml/min.[10] Separations were carried out at 25°C with an injection loop of 20 μl. The DAD detector was operated in the range of 200-650 nm, and the analysis was performed at 254 nm. Components of LI were identified by comparing their retention times and UV spectra with those of authentic standards (liquiritin and glycyrrhizin) under identical analysis conditions. Solutions at different concentrations of each standard were injected into the HPLC to check the linearity between concentration and peak areas, and a response factor was calculated. Quantifications of liquiritin and glycyrrhizin were done using these calibration factors.

### Activation of immune cells

The activation of total lymphocytes and T cells was analyzed by flow cytometry, measured by CD69 expression.[11] Using sterile 96-well flat-bottomed plates, 100 μl of peripheral blood suspension were incubated along with 100 μl of X-Vivo medium containing test samples with increasing concentrations and without test samples (control). The mitogen PHA was used as a positive control at 10 μg/ml. Samples were filtered through 0.2 μm filters before use. Final concentrations of LI and compounds in the assay media were in the range of 100-800 μg/ml and 12-100 μg/ml, respectively. Plates were then incubated at 37°C with 5% CO_2_ for 24 hours. After incubation, 40 μl of each incubated suspension was labeled with a cocktail of fluorescently labeled antibodies CD69 PE, CD69 FITC, CD3 APC. Flow cytometric analysis was performed on a Cytomics FC500 flow cytometer (Beckman Coulter, USA) and data were analyzed by CXP analysis software (Coulter Electronic, USA). Cell activation was measured by increases in mean fluorescence intensities (MFI) because of the expression of CD69 on the cell surface. Fluorescence signals were obtained as logarithmically amplified signals. Values of activation are presented as activation index (AI), which were calculated by dividing the MFI of treated cells with test samples by that of untreated cells (control). A positive immune cell response was defined as an AI ≥ 2. Data were presented as the mean ± SD for three experiments.

### Cell cycle progression of lymphocytes

To assess the effect of samples on the cell cycle progression of lymphocytes, the DNA content of individual cells stained with propidium iodide was analyzed by flow cytometry.[5] Briefly, 100 μl of diluted peripheral blood (1:10) was incubated with test samples for 72 hours. Lymphocytes were isolated from red cells by lysis in hypotonic solution followed by centrifugation. Cells were fixed with 4% paraformaldehyde for at least five minutes and the supernatant was removed by centrifugation at 1100 rpm for 10 min. A volume of 500 μl of 0.5% Tween, 50 μl of ribonuclease A, and 50 μl of propidium iodide solution were added and the mixture was incubated for 60 minutes at 37°C with 5% CO_2._ Fluorescence (DNA content) was measured on a Cytomics FC500 flow cytometer (Beckman Coulter, USA). A minimum of 10 000 cells analyzed in each sample served to determine the percentages of cells in each phase of the cell cycle; Multicycle AV software was used for the analysis (Phoenix Flow Systems Inc., USA).

### Statistical analysis

Data were analyzed by one-way analysis of variance (ANOVA) followed by Tukey's multiple comparison test using the GraphPad Prism 5 software. Differences were considered significant when *P* < 0.05.

## RESULTS

**Figure 1:**
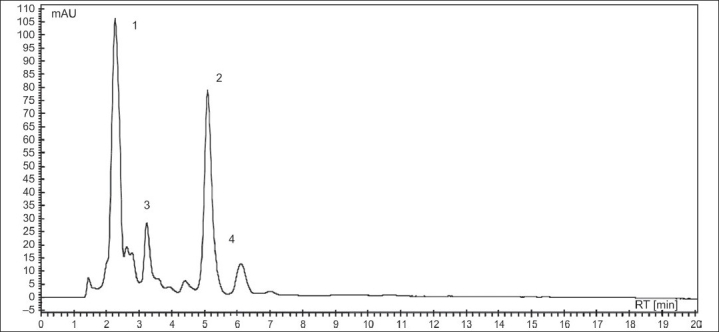
HPLC chemical profile of licorice infusion (LI). Detection at 254 nm. Peaks: (1) liquiritin; (2) glycyrrhizin; (3) and (4) glycyrrhetic acid derivatives (at column width)

**Figure 2:**
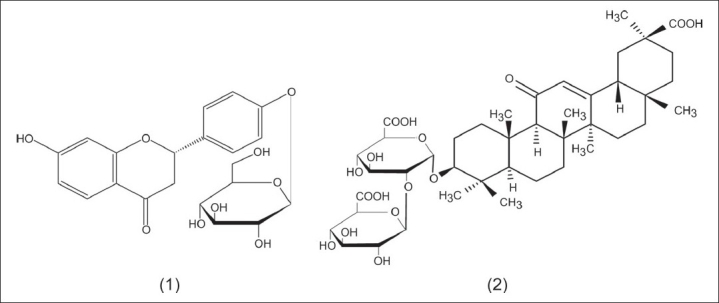
Structures of liquiritin (1) and glycyrrhizin (2) (at column width)

The present study reports the total content of phenolic, flavonoids, and tannins in a licorice infusion [Table 1]. The extraction yield of licorice lyophilized infusion (LI) was about 30%. The data from the HPLC-DAD analysis of LI is presented in Table 1, whereas, the HPLC chromatogram is presented in Figure 1. The two major components of LI were identified as liquiritin 1 and glycyrrhizin 2 [Figure 2] by comparing with the retention times and UV spectra of authentic standards. Peaks 3 and 4 of the chromatogram [Figure 1] showing UV λmax at 248 nm were preliminarily identified as derivatives of glycyrrhetic acid.

**Table 1:** Content of liquiritin, glycyrrhizin, and total polyphenolics in licorice infusion

Compounds	Content in dried roots	Content in lyophilized extract (LI)	Regression equation	Correlation coefficient (r^2^)
Liquiritin	1.55 ± 0.05	5.18 ± 0.17	Y = 246.02X + 1.9512	0.9999
Glycyrrhizin	2.23 ± 0.04	7.43 ± 0.16	Y = 161.05X + 0.0747	0.9995
TP content(a)	1.75 ± 0.02	5.83 ± 0.06	Y = 0.1011X + 0.0206	0.9992
TF content(b)	0.21 ± 0.01	0.70 ± 0.02	Y = 0.0535X + 0.0002	0.9999
TT content(c)	0.24 ± 0.01	0.78 ± 0.01	Y = 0.1011X + 0.0206	0.9992

(a) Total phenolics (TP) and

(c) total tannins (TT) contents are expressed as mg gallic acid equivalents (GAE)/100 mg LI.

(b) Total flavones and flavonols (TF) content is expressed as mg quercetin equivalents (QE)/100 mg LI.

Contents of liquiritin and glycyrrhizin are expressed as percentage (w/w) in terms of dry weight. Each value represents mean (n = 3)± SD

**Figure 3:**
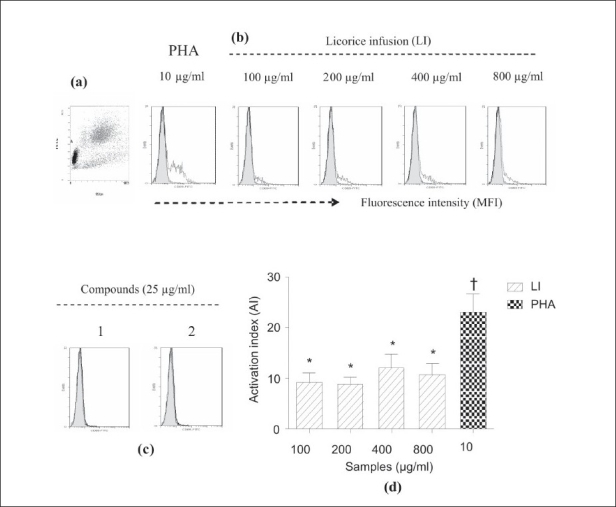
Effect of licorice infusion (LI), liquiritin (1) and glycyrrhizin (2) on activation of total lymphocytes, as measured by CD69 expression. Lymphocytes were initially gated by their characteristic forward (FSC) and side scatter (SSC) profiles, which represent size and granularity, respectively. The activated lymphocytes in circular gate A (a) were then analyzed for fluorescent intensity. The filled histograms represent the group control (untreated) and the open histograms the stimulated (treated) group (b). Compounds 1 and 2 (at range of 12-100 μg/ml) were inactive and histograms (c) are shown as representative figures. (at page width); Bar graphic (d) shows the effect of LI on the total lymphocytes as activation index (AI)

**Figure 4:**
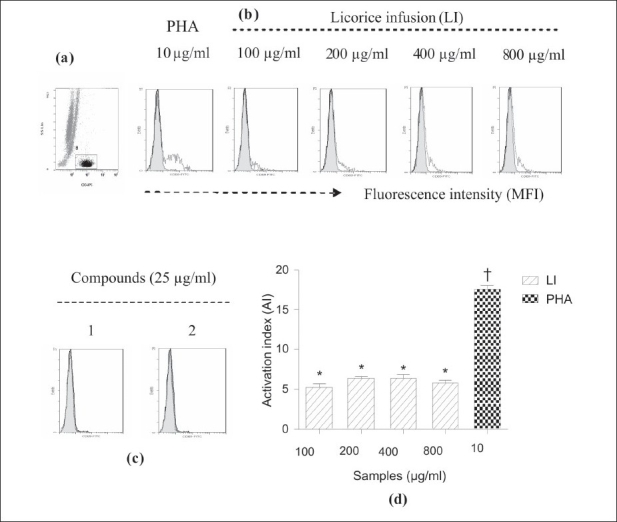
Effect of licorice infusion (LI), liquiritin (1) and glycyrrhizin (2) on activation of T lymphocytes, measured by CD69 expression. For visualization of T lymphocyte subset, the surface immunostaining with anti-human CD3 was used. The activated cells in rectangular gate B (a) were then analyzed for fluorescent intensity. The filled histograms represent the group control (untreated) and the open histograms the stimulated (treated) group (b). Compounds 1 and 2 (at range of 12-100 μg/ml) were inactive and histograms (c) are shown as representative figures. (at page width); Bar graphic (d) shows the effect of LI on the T lymphocytes as activation index (AI)

**Figure 5:**
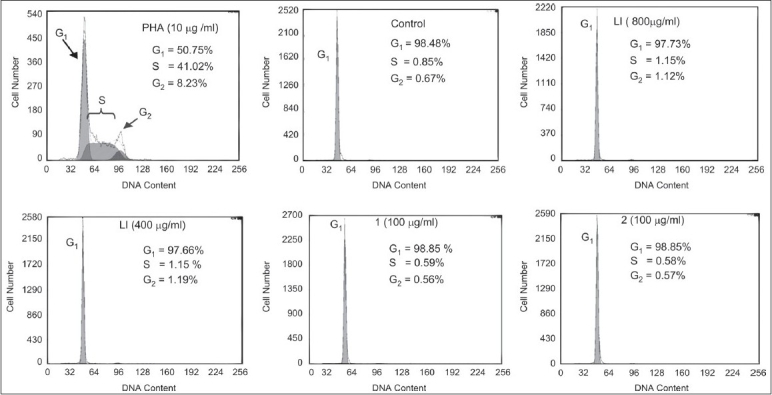
Effects of licorice infusion (LI), liquiritin (1) and glycyrrhizin (2) on the cell cycle progression of human lymphocytes. The LI, 1, and 2 showed no meaningful effect on cell cycle progression of lymphocytes. One representative experiment is shown and the percentages of cells in each phase of the cell cycle (G_1_, S, and G_2_ phases) are shown. (at column width)

The effects of LI, 1, and 2 on the activation of total lymphocytes, measured by the CD69 expression, was analyzed by flow cytometry. As indicated by a shift to the right in histograms [Figure 3b, 4b], the CD69 expression on cells was increased after 24 hours. As the cells were activated, the amount of the fluorescently labeled antibodies bound to them increased. The LI moderately stimulated the expression of CD69 on lymphocytes in a concentration-independent manner in the range of 100-800 μg/ml. Values of the activation index (AI) of total lymphocytes treated with LI in the range of 100-800 μg/ ml did not differ significantly among them (*P* < 0.05), but were lower than the AI value exhibited by cells treated with PHA [Figure 3d]. The LI showed a similar effect on T cells, but on a lower scale [Figure 4d]. Compounds 1 and 2 in the range of 12-100 μg/ml did not stimulate the CD69 expression on lymphocytes [Figure 3c, 4c]. It was observed that the LI, 1, and 2 showed no meaningful effect on cell cycle progression of lymphocytes. Most of the lymphocytes treated with LI, 1, and 2 were in G_1_ phase, and there was only a very small number of cells in S or G_2_ phases. The PHA stimulated lymphocytes to enter into the S and G_2_ phases after 72 hours of culture [Figure 5].

## DISCUSSION

### Chemical analysis

About 300 kinds of phenolic compounds have been isolated from various species of *Glycyrrhiza* and many of them are described as exhibiting biological actions that supplement the efficacy of licorice.[3] Polyphenols are bioactive compounds believed to be involved in the defence process against oxidative damage in biological systems, owing at least in part to their antioxidant properties.[12] In a prior study, the content of TP (7.42 μg/mg) and TF (0.88 μg/mg) in a licorice extract were reported.[13] In another study, the TF content of *G*. *pallidiflora* was measured by two different methods. The TF contents were 2.09 mg/100 mg and 4.27 mg/100 mg of the extract, measured by the AlCl_3_ and Al(NO_3_)_3_ methods, respectively.[14] Most reports on the flavonoid content in licorice are usually based on flavanones and chalcone such as liquiritin, isoliquiritin, and their corresponding aglycones.[3] Unlike flavanones from licorice, other types of minor flavonoids such as flavones and flavonols have been shown to exhibit strong antioxidant effects.[15] To date, there is very limited information on the TT content in the roots of *Glycyrrhiza* species. Two flavonoids, named licochalcone B and glycyrrhisoflavone, were isolated from an acetonic- aqueous extract of licorice and they exhibited tannin-like activity.[15] Glycyrrhizin has been reported to constitute 10-25% of licorice extract[16] and the liquiritigenin glycosides were reported to constitute 1.6% of licorice aqueous extract.[17] As can be noticed, the comparison between the values reported by different laboratories can be complicated because of substantial differences in sample preparation, geographic sources, harvesting, and expression of results.

### Activation of immune cells

The CD69 glycoprotein is a very early cell activation molecule expressed on the surface of T, B, and Natural Killer (NK) cells following activation.[18] It can appear within 1-2 hours of activation and exhibits maximal expression levels between 18 and 24 hours after stimulation.[19] Although a physiological ligand for CD69 has not yet been identified, its wide distribution and the observation that crosslinking of the molecule generates intracellular signals suggest a significant role for CD69 in immune response.[20] Three major types of lymphocytes include T, B, and NK cells. Adaptive immune responses are based on the activities of T lymphocytes (also called T cells) and B lymphocytes (B cells). The key to the adaptive immune system is the presence of the extremely variable antigen-specific receptors of the T lymphocytes (called TCRs) and B lymphocytes (called B-cell receptors [BCRs], immunoglobulins, or antibodies). NK cells are part of the lymphoid lineage but are distinct from T and B lymphocytes because they do not express the specialized receptors associated with the adaptive immune response. Instead they have two other types of receptors that determine their ability to identify and kill targeted host cells: Killer activation receptors and killer inhibition receptors. NK cells are important elements in innate defenses against virally infected and cancerous host cells.[21]

As can be deducted from AI values [Figure 3d, 4d], the activation of total lymphocytes and T cells by LI represents about 50 and 34% of those promoted by PHA, respectively. In addition, by comparing the AI of total lymphocytes with those of T cells, it was observed that about 50% of the activation of total lymphocytes could proceed from the T cells activation. Although the effect of LI on the CD69 expression was moderate, this fact could be hypothetically interpreted as beneficial, given that increased level of CD69 expression on T cells has been associated with some autoimmune diseases.[22,24] Licorice has multiple constituents and not one active ingredient; therefore, it is possible that they are able to act in a regulatory way, both activating and modulating the immune response. It is also questionable whether the LI immunostimulating effect is long-term because the CD69 expression on the surface of immune cells not only occurs very rapidly, but also declines rapidly (disappearing after two days). Therefore, this study measured the CD25 expression, a later-expressed activation marker, but the expression was very low (data are not shown). Apparently, regular consumption of licorice infusion would be important for its long-term effect.

Recently, tinctures of *Echinacea purpurea*, *Astragalus membranaceus*, and *G*. *glabra* were shown to stimulate T cells, determined by CD69 expression. These three herbs had an additive effect on CD69 expression when used in combination and no chemical compound was related with the effects observed.[11] In contrast, previous *in vitro* studies revealed antiviral activity of glycyrrhizin against HIV-1, SARS related coronavirus, respiratory syncytial virus, arboviruses, vaccinia virus, and vesicular stomatitis virus. Mechanisms for antiviral activity included the reduced transport to the membrane and sialylation of viral surface antigen, reduction of membrane fluidity leading to inhibition of fusion of the viral membrane with the cell, induction of interferon gamma in *t*-cells, inhibition of phosphorylating enzymes in vesicular stomatitis virus infection, and reduction of viral latency.[4]

Licorice is popularly consumed in the form of teas and infusions;[6] however, studies on the immunomodulating effects of licorice by measuring the CD69 expression have been focused mainly on its tincture.[11] Infusion and tincture of licorice have different chemical profiles[23,25] and therefore are expected to show different bioactivities. In the present study, the effect of a LI on the activation of lymphocytes was investigated for the first time. In contrast to a number of prior investigations,[26] the current study indicates that glycyrrhizin does not seem to be involved in the immunostimulating effect of LI, as measured by the CD69 expression.

### Cell cycle progression of lymphocytes

The immunomodulating action of herbs may involve not only cell activation, but also cell proliferation. It is described that the interaction of lymphocytes with antigens or PHA initiates a cascade of biochemical events and gene expression, which induces the resting immune cells to enter the cell cycle, and then begin proliferating and differentiating.[27] By measuring the incorporation of propidium iodide in lymphocyte DNA using flow citometry, it was observed that LI, 1, and 2 had no effect on the cell cycle progression of lymphocytes. It has been suggested in different systems that CD69 expression may precede cell proliferation, maturation or differentiation.[28] Nevertheless, the CD69 expression may not completely overlap with cells that undergo DNA synthesis following antigen receptor engagement. Although the immunologic consequences of lymphocyte clonal expansion are well established and critical to the development of immunity, the relevance of CD69 expression to this process is less understood.[29] The emerging data from the present study suggests that the LI-activated lymphocytes express CD69 as a result of a proliferation-independent process. In the current investigation, the stimulating effect of LI and its two major constituents on the cell cycle progression of lymphocytes was investigated for the first time.

## CONCLUSION

The present *in vitro* study demonstrates that licorice infusion (LI) stimulates the activation of lymphocytes, mainly T cells. The two major components of LI were identified as liquiritin (1) and glycyrrhizin (2) and they seem not to be involved in the activation of lymphocytes. Flavones and flavonols were identified as minor constituents of LI. This report establishes the need for future studies to determine the *in vivo* effect of LI on lymphocytes and to identify its active constituentes. This herbal preparation might be a potential alternative for mounting an effective immune response and as a preventive barrier against both viral and bacterial infections, and during immunosenescence.
